# Monitoring Bone Density Using Microwave Tomography of Human Legs: A Numerical Feasibility Study

**DOI:** 10.3390/s21217078

**Published:** 2021-10-26

**Authors:** Mohanad Alkhodari, Amer Zakaria, Nasser Qaddoumi

**Affiliations:** 1 Department of Electrical Engineering, American University of Sharjah, Sharjah P.O. Box 26666, United Arab Emirates; aszakaria@aus.edu (A.Z.); nqaddoumi@aus.edu (N.Q.); 2 Department of Biomedical Engineering, Khalifa University, Abu Dhabi P.O. Box 127788, United Arab Emirates

**Keywords:** microwave tomography, two-dimensional imaging, electromagnetic signals, finite-element method, contrast-source inversion, bone imaging, bone density, bone volume fraction

## Abstract

A major cause of bone mass loss worldwide is osteoporosis. X-ray is considered to be the gold-standard technique to diagnose this disease. However, there is currently a need for an alternative modality due to the ionizing radiations used in X-rays. In this vein, we conducted a numerical study herein to investigate the feasibility of using microwave tomography (MWT) to detect bone density variations that are correlated to variations in the complex relative permittivity within the reconstructed images. This study was performed using an in-house finite-element method contrast source inversion algorithm (FEM-CSI). Three anatomically-realistic human leg models based on magnetic resonance imaging reconstructions were created. Each model represents a leg with a distinct fat layer thickness; thus, the three models are for legs with thin, medium, and thick fat layers. In addition to using conventional matching media in the numerical study, the use of commercially available and cheap ultrasound gel was evaluated prior to bone image analysis. The inversion algorithm successfully localized bones in the thin and medium fat scenarios. In addition, bone volume variations were found to be inversely proportional to their relative permittivity in the reconstructed images with the root mean square error as low as 2.54. The observations found in this study suggest MWT as a promising bone imaging modality owing to its safe and non-ionizing radiations used in imaging objects with high quality.

## 1. Introduction

Osteoporosis is a disease that causes bone to lose its strength and mass. According the National Osteoporosis Foundation (NOF), more than 33.6 million people are suffering from low bone density of the hip due to this disease [1]. The main cause of osteoporosis is Vitamin D deficiency, which is crucial for bones to absorb the calcium supplied by the body to stay healthy and in good shape and strength [2,3]. In the United Arab Emirates, where this research was conducted, it is estimated that more than 78% of the population suffers from Vitamin D deficiency [4], which has led to serious problems. Many factors can result in Vitamin D deficiency, including obesity, genetics, and cultural dress codes.

Therefore, frequent checkups and optimum diagnosis are needed to resolve this major issue efficiently. The current gold standard in diagnosing osteoporosis is the dual-energy X-ray absorptiometry (DXA) and the quantitative computed tomography (QCT). X-ray used in these techniques is capable of providing an accurate measure for the overall bone mineral density and, thus, an evaluation for both health and strength [5].

Despite its efficiency in bone diagnostics, it uses excessive radiations that could be harmful for frequent bone monitoring and assessment applications [5,6]. In addition, magnetic resonance imaging (MRI) has been used for osteoporosis evaluation [7,8,9,10]. However, MRI may be expensive as a public health service, its equipment is bulky for timely and frequent measurements, and it requires a long time for scanning the object-of-interest (OI). Therefore, there is a need for a safer, simpler, faster, and cheaper imaging modality that is capable of detecting bones under various mass conditions. Examples of such modalities are ultrasound [11], electric impedance tomography (EIT) [12], and microwave tomography (MWT), which is the focus of the work presented herein.

Recently, microwave tomography (MWT) has emerged in many biomedical imaging areas, including breast and lung cancer detection [13,14] and brain stroke analysis [13]. An important advantage in MWT is the utilization of non-ionizing radiations in a form of electromagnetic waves to image the OI [15,16]. In MWT, the OI is initially radiated through several antennas acting as transmitters with low-power electromagnetic signals, and then the same antennas act as receivers to acquire the scattered signals due to the presence of this OI in the imaging chamber [13].

The collected data are used within an optimization algorithm to reconstruct tomographic images of the OI in a form of two-dimensional (2D) slices or color maps. The reconstruction of images involves mathematical calculations for the electrical properties of different tissues in the OI, including the relative permittivity (dielectric constant) and effective conductivity.

In the literature, several studies have shown a strong association between the bone volume fraction (BVF), which is a metric that shows bone strength, mass, and quality, and the corresponding electrical properties [17,18]. BVF is a measurement for the volume of mineralized bone per unit volume of the selected OI [19]. In Meaney et al. [17], they demonstrated that bone’s relative permittivity values estimated using microwave tomography increase with a loss in bone mass. In addition, Refs. [6,20,21] concluded a strong potential of MWT in imaging bones of the calcaneus (heel bone).

In many of these studies, researchers prefer selecting regions of cortical bones over the trabecular (spongy) bones for the stiffness and lesser inclusion of fluids, which makes image reconstruction more efficient. All of the aforementioned studies, however, did not examine MWT images and systems for monitoring bone variations as changes in image reconstruction values. Therefore, further studies should be conducted to evaluate the screening of bones in MWT under variations in bone density.

In this paper, a numerical feasibility study was conducted to evaluate the efficiency of monitoring bone density variations, as changes in BVF values in MWT reconstructed images. The novelty of the proposed work lies in utilizing MWT reconstructions, which shows a map of dielectric properties of objects, to observe changes in bone mass. Unlike the current state-of-the-art studies, the proposed approach relies on an in-house finite-element contrast source inversion (FEM-CSI) algorithm [22,23] that allows enhancing the reconstructions even further by incorporating prior information about the structure and dielectric properties of tissues, varying the shape of the selected imaging domain to reduce complexity and to utilize variable locations for antennas.

In addition, this study proposes for the first time the use of a commercially-available ultrasound gel for the use of MWT imaging. This allows for the development of user-friendly systems that could be wearable and do not require bulk configuration with the conventional glycerin/water-based systems. In addition, the proposed study was designed based on 2D MWT configuration; thus, it reduces the computational complexity often found in 3D systems modeling. Moreover, this study was carried out using anatomically-realistic models of human leg under three fat layer thickness scenarios, namely thin, medium, and thick fat; this allows study of the impact of fat thickness variations on the reconstructed images.

The models were created using MRI reconstructions. Further, in imaging the leg, the midsection of the lower leg was selected due to its accessibility, with the ability to position the antenna array of a MWT system around it easily, while, at the same time, it includes mostly cortical (stiff) bones. In addition, for wearable MWT configuration, the leg would be a more suitable part to place antennas, especially when frequent monitoring of bones is needed. It should be noted that preliminary investigations related to this work have been presented in [24,25,26]. The proposed feasibility study sets the base towards designing and implementing real-life MWT systems for bone imaging.

## 2. Materials and Methods

### 2.1. Generation of Anatomically-Realistic Models and the Forward Problem

#### 2.1.1. Numerical Models of Human Leg

The first step of the study was to generate the numerical models that were utilized. Furthermore, three anatomically-realistic models of human leg were generated based on magnetic resonance imaging (MRI) cross-sectional images. Each MRI image represents a certain fat scenario, including thin, medium, and thick fat layers. These images were obtained from online databases [27,28,29], respectively. Different fat thicknesses were selected to understand the effect on detecting the bones and estimating their electrical properties.

The generation of each scenario’s model was performed using GMSH software [30] to create a complete triangular mesh whose boundary points were extracted directly from the MRI images in MATLAB. Using MATLAB, data points were extracted from skin, fat, muscle, and bones (tibia and fibula) tissue boundaries. Each tissue was represented in GMSH as a surface tissue, and each surface includes a finite amount of triangular points and shapes, where all computations are going to take place. In the final model, the model is usually surrounded by an imaging domain that forms the boundary of calculations.

In the generated mesh, the characteristic length (which is the length of a mesh triangle’s side) is smaller than λ/10, where λ is the wavelength calculated as,

(1) $$\lambda =c/\left(f\sqrt{\epsilon_{r,\max}}\right).$$

**Figure 1:**
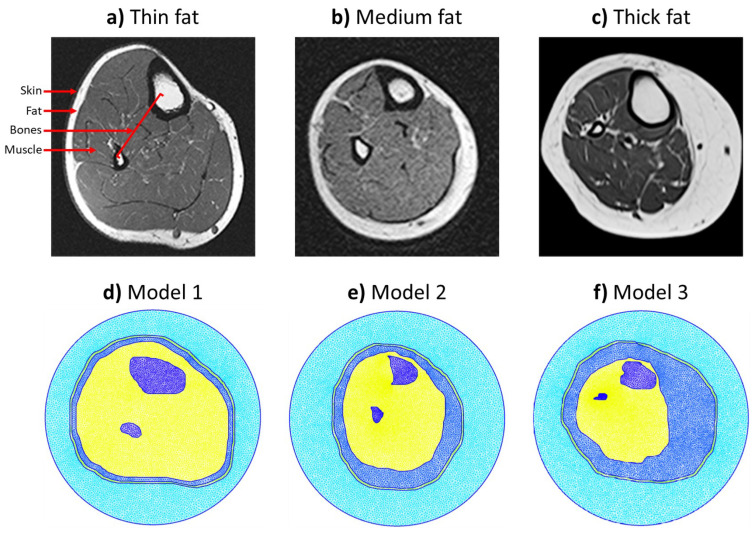
Generation of the three cross-sectional MRI-based anatomically-realistic leg models representing three fat thickness scenarios: (**a**,**d**) Thin fat model 1 [27], (**b**,**e**) Medium fat model 2 [28], and (**c**,**f**) Thick fat model 3 [29].

Here, $c=3\times 10^{8}$ m/s is the speed of light in free-space, *f* is the simulation frequency, and $\epsilon_{r,\max}$ is the largest possible value of the dielectric constant within the model. The finite-element meshes for each model are shown in Figure 1. It is recommended to have small length of a mesh triangles so that it captures small details in tissues when the calculations take place. As an example, Model 1 had a total of 254,044 triangles supported by 127,153 nodes.

#### 2.1.2. Solving the Forward Problem in MWT

The forward problem of MWT systems requires calculations for the electric fields values at the receiving antennas after transmitting low-power electromagnetic signals by the sources. To achieve this, the FEM solver developed by A. Zakaria et al. [31] was used with inputs as follows.

**Figure 2:**
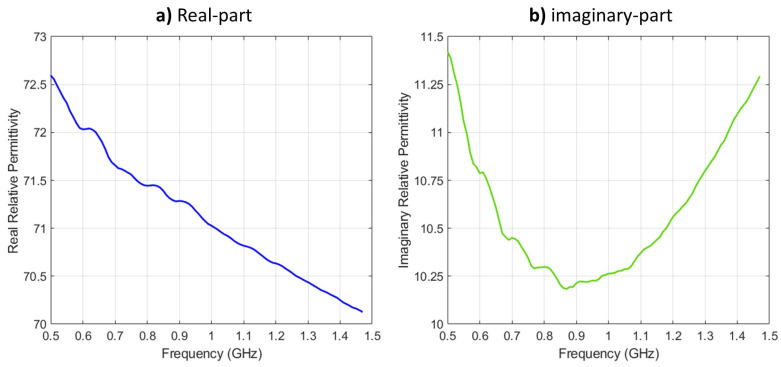
Relative complex permittivity (real and imaginary) of AquaSonic 100 gel: (**a**) real-part and (**b**) imaginary-part.

- *Numerical model*: 2D triangular mesh (discussed in Section 2.1.1).
- *Operating Frequency*: 0.8 GHz.
- *Transmitters’ (TX) and receivers’ (RX) configuration*: 24 point-sources (TX) and 24 point-sinks (RX) collocated and equally distributed around the model outer most layer.
- *Matching medium surrounding the OI*: 80:20 glycerin/water solution with relative complex permittivity of $\epsilon_{r}=26-j18$ [32,33] and a commercially-available ultrasound gel (dielectric properties shown in Figure 2).
- *Relative complex permittivity of various tissues in the model*: given in Table 1 and based on [6,34].

**Table 1:** Bulk electrical properties in the human leg model at 0.8 GHz.

Region	Relative Complex Permittivity ($\epsilon_{r}$)
Skin	42−j18.8
Fat	11−j2.3
Muscles	55−j20.5
Healthy Bone	13−j3.0

**Figure 3:**
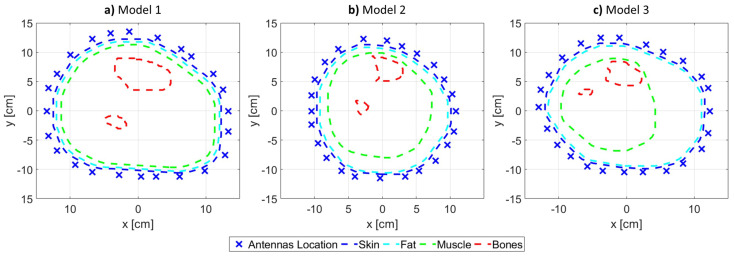
The problem configuration for each model, which includes various tissue boundaries alongside the location of the antennas: (**a**) Model 1, (**b**) Model 2, and (**c**) Model 3.

The problem configuration is shown in Figure 3, which includes the antenna locations, the imaging domain boundary, and various tissue boundaries. The tissue boundaries are used as input to GMSH for mesh generation. Moreover, the meshes alongside the antenna location were input to the FEM solver for electric field calculations. The objective of the forward problem was to simulate an actual MWT system (close to being wearable) and calculate the electric field values at the receivers.

The effect of having a smaller imaging domain as well as close-to-skin antenna locations was presented in [25], where it was shown that this enhances the reconstruction of tissues. The selection of the operating frequency (0.8 GHz) was to ensure that antennas are of a reasonable size (the smaller the frequency, the larger the antenna) as well as to ensure enough penetration for electromagnetic signals inside the leg (the larger the frequency, the smaller the signal penetration, and the higher the propagation losses).

#### 2.1.3. Utilization of an Ultrasound Gel as a Matching Medium

In addition to using the regular glycerin/water mixture for a matching medium in the MWT system, this work introduces the ultrasound gel as a promising matching medium for MWT applications. The gel is commonly used for portable imaging technique such as ultrasound and echocardiography for its aqueous, bacterio-static, non-sensitizing, and non-irritating nature. The world standard for ultrasound gels is the AquaSonic 100 [35]. To the best of the authors’ knowledge, there have been no previous studies on the use of AquaSonic 100 ultrasound gels in MWT.

The electrical properties (real and imaginary relative permittivity) of the gel were measured because they were not found in literature. They were measured using Keysight’s N1501A Coaxial Dielectric Probe [36]. The electrical properties of the ultrasound gel are shown in Figure 2. From the figure, at 0.8 GHz frequency, the complex relative permittivity is $\epsilon_{r}=71.4-j10.3$, and this was selected for evaluating the forward and inverse problems in MWT. Such values were expected since the gel is water-based.

### 2.2. Image Reconstruction and the Inverse Problem

All synthetic data were collected by the receivers for each of the three different models and used as inputs to the inversion algorithm for image reconstruction. The inverse problem was solved within the framework of FEM and multiplicatively-regularized contrast source inversion (CSI) method [22,37].

In FEM-CSI, the objective is to minimize the following cost functional:

(2) $$F^{\mathrm{CSI}}\left({\underline{w}}_{t},\chi \right)=F^{S}\left({\underline{w}}_{t}\right)+F^{D}\left({\underline{w}}_{t},\chi \right),$$

where

(3) $$F^{S}\left({\underline{w}}_{t}\right)=\frac{\sum_{t=1}^{T}{∥{\underline{u}}_{t}-M_{S}L\left[{\underline{w}}_{t}\right]∥}_{S}^{2}}{\sum_{t=1}^{T}{∥{\underline{u}}_{t}∥}_{S}^{2}},$$

and

(4) $$F^{D}\left({\underline{w}}_{t},\chi \right)=\frac{\sum_{t=1}^{T}{∥\underline{\chi }⊙{\underline{E}}_{\mathrm{inc},t}-{\underline{w}}_{t}+\underline{\chi }⊙M_{D}L\left({\underline{w}}_{t}\right)∥}_{D}^{2}}{\sum_{t=1}^{T}{∥\underline{\chi }⊙{\underline{E}}_{\mathrm{inc},t}∥}_{D}^{2}}.$$

In (3) and (4), the discretized variables and operators are:

- ${\underline{u}}_{t}\in C^{R}$ is a vector of size *R* of the scattered electric field values measured at the receivers for a transmitter *t*. The receivers are located on measurement surface *S*.
- ${\underline{w}}_{t}\in C^{N}$ is a vector of size *N* for the contrast source values inside the imaging domain *D* for a transmitter *t*.
- ${{\underline{E}}^{I}}_{\mathrm{inc},t}\in C^{I}$ is a vector of size *I* for the incident field values inside the imaging domain *D* for transmitter *t*.
- $\underline{\chi }\in C^{I}$ is a vector of size *I* for the contrast values inside the imaging domain *D*.
- $L\in C^{N\times N}$ is an FEM matrix operator. This operator contains information about the problem’s boundary and background (whether it is homogeneous or inhomogeneous).
- $M_{S}\in C^{R\times N}$ is a matrix operator that calculates the scattered field values at the receiver locations on measurement surface *S*.
- $M_{D}\in C^{I\times N}$ is a matrix operator that calculates the scattered field values inside the imaging domain *D*.

Furthermore, in (3) and (4), ${∥\cdot ∥}^{2}$ is $L_{2}$-norm or Euclidean norm, and the operator ⊙ is element-wise vector multiplication. At each iteration of the FEM-CSI algorithm, the two variables $\underline{\chi }$ and ${\underline{w}}_{t}$ are updated successively. The contrast source variable ${\underline{w}}_{t}$ is updated using a conjugate-gradient method with Polak–Ribière search directions. Next, the contrast variables $\underline{\chi }$ are updated analytically. These two variables are updated until the algorithm converges. The details of the FEM-CSI algorithm as well as multiplicative regularization are outlined in [31].

### 2.3. Detection of Bone Density Variations

In this section, the focus is on the ability to detect variations in bones’ electrical properties due to changes in the bone density. As discussed in the introduction, current gold standard techniques to evaluate bones mass is the BVF metric, which is a representation of the overall volume of bone within an OI. The changes of bone mass and BVF can result in changes in the relative permittivity values estimated using MWT, and thus, lead to variations in the reconstruction of images.

For each leg model, synthetic data were collected for five different bone health scenarios. In each scenario, the bones’ electrical properties are estimated as follows:

- Bones with 0.50 BVF: $\epsilon_{r}=13-j3.0$,
- Bones with 0.45 BVF: $\epsilon_{r}=14-j3.05$,
- Bones with 0.35 BVF: $\epsilon_{r}=16-j3.1$,
- Bones with 0.25 BVF: $\epsilon_{r}=18-j3.2$,
- Bones with 0.10 BVF: $\epsilon_{r}=23-j3.4$.

These values were calculated from a study conducted in [17], where bones with 0.5 BVF represents healthy bones case and a 0.1 BVF represents a severe bone mass loss.

#### Expert-Eye Localization of Bones

A simple algorithm was developed to evaluate bones by extracting their electrical properties from the inversion results (image reconstructions). The algorithm is called “expert-eye localization”, as it was imagined that an experience radiologist would look at the MWT images for bone location identification. In this algorithm, the regions-of-interest (ROIs) of bones were manually determined and outlined using the imaginary part of the relative permittivity images.

These extracted regions were mapped on the real part relative permittivity images to extract masks with corresponding values of bones. To ensure not to include any errors or non-bone regions, regions that were more than the first quartile of the ROIs’ mean dielectric constant were discarded. At the end, the remaining values on the mask were used to determine the average value of permittivity at different bone mass scenarios.

## 3. Results

**Figure 4:**
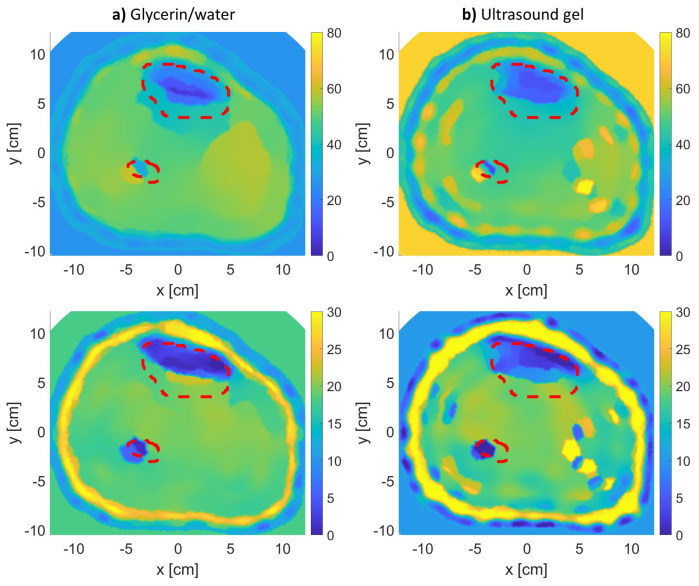
The real (**top row**) and imaginary (**bottom row**) relative complex permittivity results for leg model 1: (**a**) using glycerin/water matching medium, and (**b**) using the ultrasound gel matching medium. The red-dotted lines indicate the bones’ actual locations.

**Figure 5:**
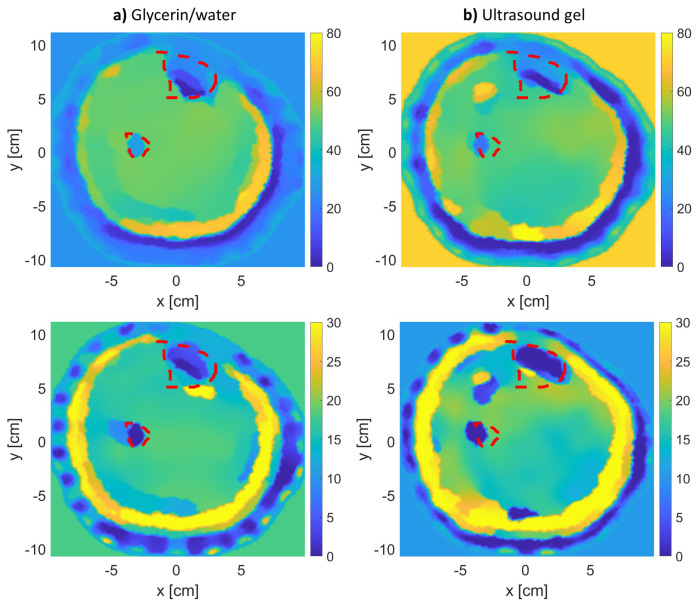
The real (**top row**) and imaginary (**bottom row**) relative complex permittivity results for leg model 2: (**a**) using glycerin/water matching medium, and (**b**) using the ultrasound gel matching medium. The red-dotted lines indicate the bones’ actual locations.

**Figure 6:**
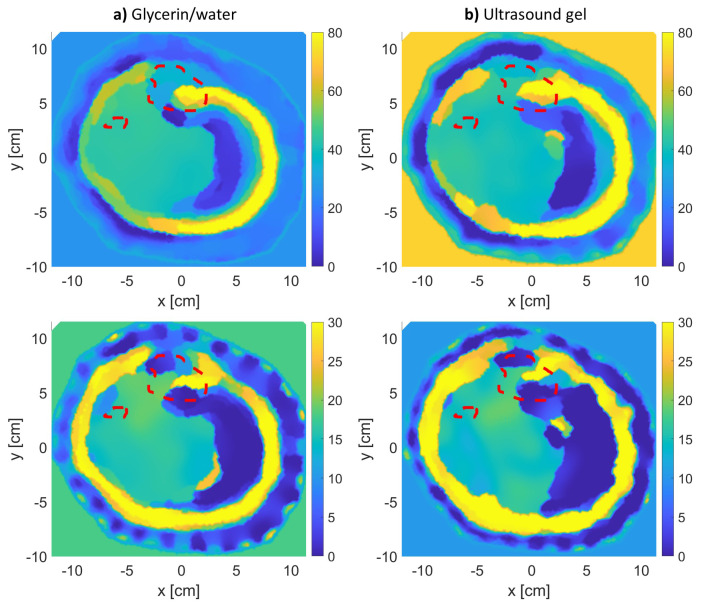
The real (**top row**) and imaginary (**bottom row**) relative complex permittivity results for leg model 3: (**a**) using glycerin/water matching medium, and (**b**) using the ultrasound gel matching medium. The red-dotted lines indicate the bones’ actual locations.

The results of the MWT inverse problem solution are shown in Figure 4, Figure 5 and Figure 6 for leg models 1–3, respectively. In producing the reconstruction results, the inversion algorithm imaging domain was only be the region with the leg, as the antennas are located to span the leg’s outer contour as shown in Figure 3. Moreover, for each model, the imaging was repeated twice: once using glycerin/water matching medium, and a second time using ultrasound gel as matching medium.

The figures for models 1 and 2 clearly show an accurate reconstruction of all four tissue layers in the leg model. The ultrasound gel returned comparable results to the regular glycerin/water solution, which suggests it to be a promising matching medium for future MWT applications. Nevertheless, using both matching medium results in the correct localization of tissues, especially for bones (tibia and fibula) as shown within the red-dotted lines. Each MWT image is a reconstruction of the dielectric properties of tissues; therefore, it is considered promising to observe true boundaries for skin, fat, and muscle tissues.

In the model 3 reconstructions (Figure 6), the inversion algorithm did not produce a satisfactory result. The thickness of the fat attenuated the scattered fields from the bones, making it difficult for the inversion algorithm to utilize these attenuated fields to reconstruct the bones.

**Figure 7:**
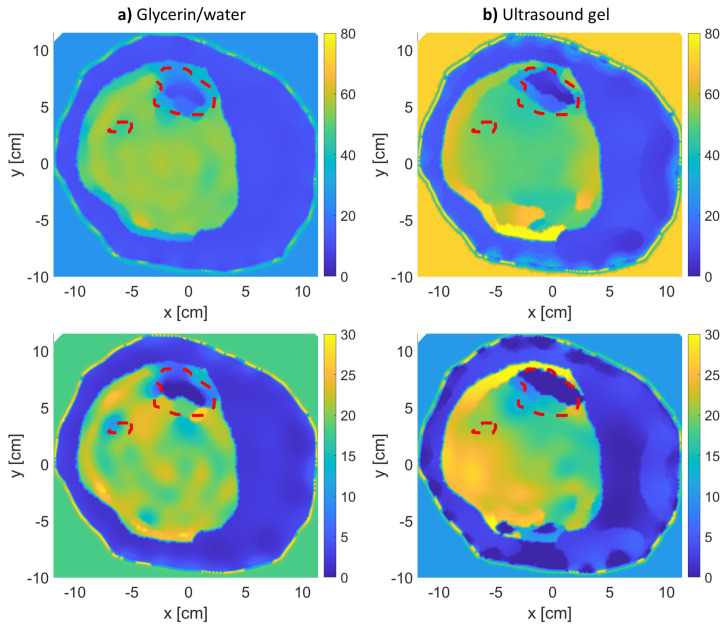
The real (**top row**) and imaginary (**bottom row**) relative complex permittivity results for leg model 3 with the incorporation of prior information about the fat layer for two different matching media: (**a**) glycerin/water solution and (**b**) ultrasound gel.

This issue can be mitigated by including the fat layer as prior information in inhomogeneous background as done in [38]. Further, model 3 reconstructions were repeated again with the actual fat layer included as inhomogeneous background. The reconstruction results for the two different matching media are shown in Figure 7. It is clear from the results that the tibia bone was well-estimated. Although the results improved, it should be noted that, in real-life scenarios, the accurate fat thickness cannot be known prior unless an MRI image is available of that region of the leg. Furthermore, to incorporate prior information, the fat thickness around the leg can be estimated using numerical techniques as done in [39] or utilizing other modalities as done using radars in [40] and using acoustic signals in [41].

**Figure 8:**
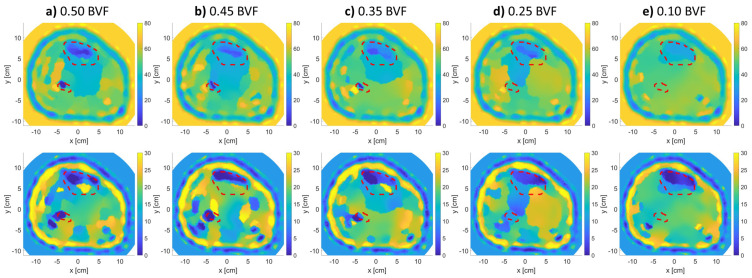
Relative complex permittivity reconstructions for leg model 1 with varying BVFs (**top row**: real, **bottom row**: imaginary). The red-dotted lines indicate the bones’ actual locations.

**Figure 9:**
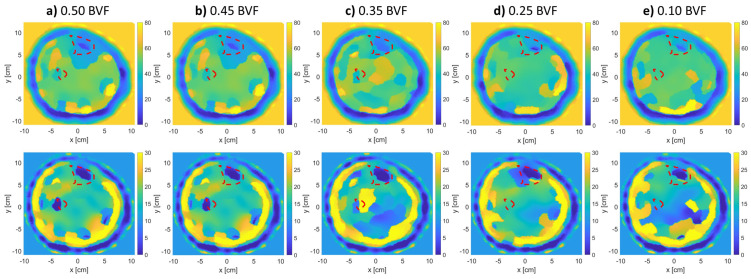
Relative complex permittivity reconstructions for leg model 2 with varying BVFs (**top row**: real, **bottom row**: imaginary). The red-dotted lines indicate the bones’ actual locations.

Further, Figure 8 and Figure 9 show the effect of changing BVF levels on the reconstructed images of models 1 and 2. Model 3 was not considered in this step due to the bad reconstruction that took place due to the thick fat layer as previously mentioned. From the figures, as the density of the bones decreases, their estimated relative permittivity values (real and imaginary) increase. This means that the bone regions start merging with the muscle regions of higher permittivity values. The tibia and fibula bones were detectable for up to 0.35 BVF. At the 0.25 and 0.1 BVF (severe) scenarios, the fibula bone was not detectable. However, an expert eye could localize the bigger bone (tibia) from the real and imaginary parts of the relative permittivity reconstructions.

**Figure 10:**
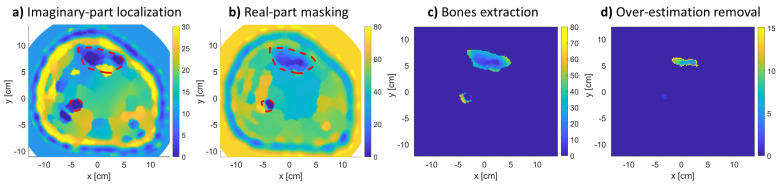
Expert-eye localization procedure followed to extract bone regions: (**a**) imaginary-part localization, (**b**) real-part masking, (**c**) extracted segments, and (**d**) over-estimated values removal.

To analyze the permittivity values of bone regions, Figure 10 shows the procedure of expert-eye localization. First, the procedure starts with localization bones manually from the imaginary-part reconstructed image of the relative complex permittivity. The selection of the imaginary part was due to the better reconstruction of tissues shape that were usually exhibited more than in the real-part image. However, the reconstruction in terms of permittivity values was better in the real-part image more than the imaginary-part one, which tended to slightly over-estimate values.

Thus, the real-part image was masked with the selected region to extract bone regions. Since the extraction is done manually and since the mask may contain muscle tissues surrounding the bones, values within the regions that are more than the first quartile of the regions’ mean dielectric constant were discarded. Finally, the mean of the remaining values was calculated and is presented as an estimation of the bones’ real relative permittivity.

**Figure 11:**
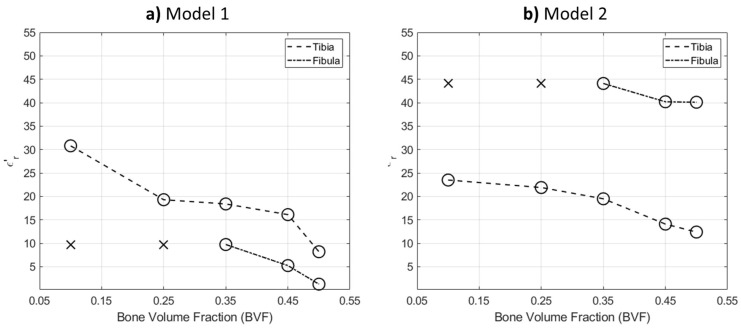
Line plot of the real part relative permittivity mean value of extracted bones: (**a**) model 1 and (**b**) model 2.

Figure 11 shows the extracted values using the expert-eye localization approach. The (x) sign refers to non-detectable bone, while the (o) sign refers to correctly localizing the bone. From the figure, the incremental pattern of the real-part permittivity is clearly shown as the BVF decreases. In model 2 reconstructions, the fibula bone estimated values were higher than the actual values; nevertheless, its shape reconstruction was satisfactory. This can be attributed to the effect of fat thickness in estimating bones’ electric properties.

### 3.1. Additional Numerical Simulations

#### 3.1.1. Additional BVF Scenarios

In addition to the BVF experimental calculations obtained from [17], we tested our algorithm with extra BVF scenarios on a 0.05 difference step. The extra BVF scenarios relative permittivity values were interpolated from [17] and represented as follows,

- Bones with 0.50 BVF: $\epsilon_{r}=13-j3.0$.
- Bones with 0.45 BVF: $\epsilon_{r}=14-j3.05$.
- Bones with 0.40 BVF: $\epsilon_{r}=15-j3.075$.
- Bones with 0.35 BVF: $\epsilon_{r}=16-j3.1$.
- Bones with 0.30 BVF: $\epsilon_{r}=17-j3.15$.
- Bones with 0.25 BVF: $\epsilon_{r}=18-j3.2$.
- Bones with 0.20 BVF: $\epsilon_{r}=19.5-j3.275$.
- Bones with 0.15 BVF: $\epsilon_{r}=21-j3.35$.
- Bones with 0.10 BVF: $\epsilon_{r}=23-j3.4$.

**Figure 12:**
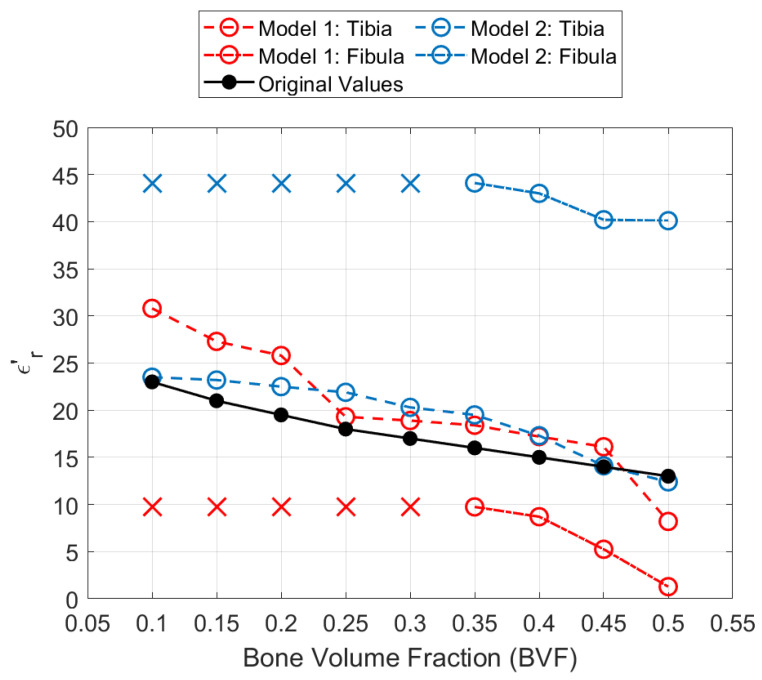
Line plot of the real part relative permittivity mean value of extracted bones under additional BVF scenarios for model 1 (red lines) and model 2 (blue lines). The black line represents the actual values of the real part relative permittivity.

Figure 12 shows the performance of the expert-eye localization detection for each of the original and extra BVF scenarios. It can be seen the the reconstructions followed an inversely proportional trend where there is an increase in the real relative permittivity values as the BVF decreases. The algorithm successfully detected such small variations in relative permittivity values; however, they were not identical to the actual ones. As shown in Table 2, the reconstruction values for the the tibia bone are close to the actual ones with a root mean square error (RMSE) of 4.52 and 2.54 for model 1 and model 2, respectively. Furthermore, in both models, the fibula bones were not detectable ((x) sign) below 0.35 BVF. Moreover, for the detected BVF scenarios, the relative permittivity values for the fibula bone were not accurately estimated with RMSE values of 8.56 for model 1 and 27.36 for model 2.

#### 3.1.2. Varying Number of Antennas

**Figure 13:**
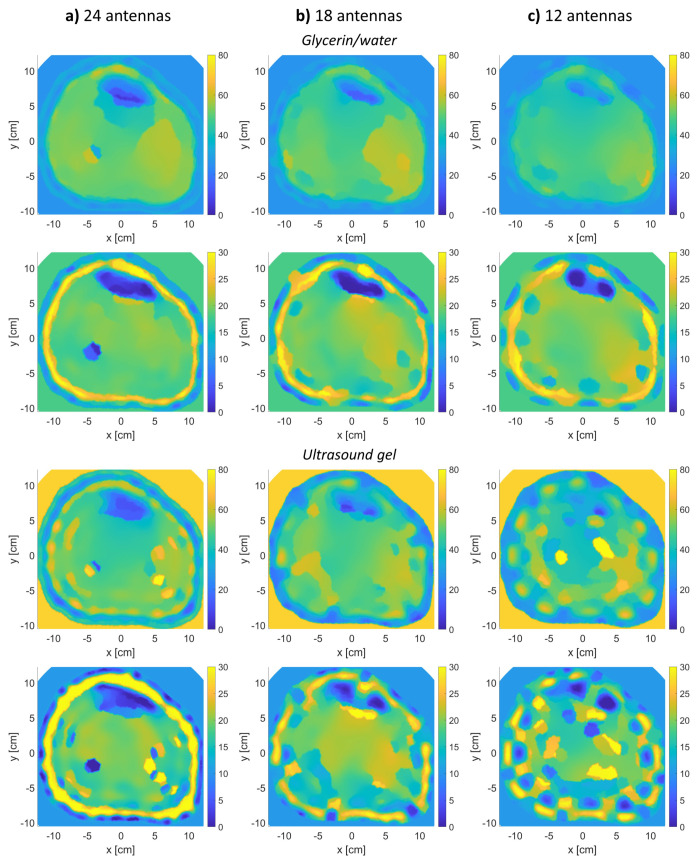
Varying the number of antennas in the reconstruction of leg model 1 using glycerin/water and ultrasound gel matching media: (**a**) twenty-four antennas, (**b**) eighteen antennas, and (**c**) twelve antennas (**top row**: real relative permittivity, **bottom row**: imaginary relative permittivity).

The proposed method was tested by varying the number of antennas. Figure 13 shows the algorithm’s reconstructions of model 1 when varying the number of antennas to eighteen and twelve (half the original number, twenty-four). The reconstructions were performed using two matching media. As shown in Figure 13, the reconstruction performance slightly changed when using eighteen antennas (Figure 13b), as tissue regions start to get blurry with less detection of the true boundaries between the various bulk tissues.

Furthermore, a minimal difference was observed when reducing the antennas even further to twelve using the glycerin/water mixture, but the reconstructions using the ultrasound gel were not as well. Using the ultrasound gel along with twelve antennas, the image reconstructions were not good as the boundaries of the various tissues were not detected well. In both antennas scenarios and using both matching media, the fibula bone was not detected; this was not the case when twenty-four antennas were used.

## 4. Discussions

### 4.1. MWT as a Promising Imaging Modality

In this work, a MWT system for monitoring bone density variations was developed using numerical simulations. The proposed system can detect variations in the bone density by estimating the electrical properties of bones. The proposed system utilizes safe non-ionizing electromagnetic radiation to image the human legs; thus, scans on patients can be performed more frequently to monitor variations in bone densities. The proposed system can be made portable, allowing for use by patients at their homes.

The proposed system uses safe and non-harmful ultrasound gel as matching medium, which is easy to clean and store. The proposed study paves the way towards implementing a real-life MWT system, which can serve as a suitable wearable system for in-house bone monitoring applications. The ability to use numerical simulations as represented in this study allows for evaluating different scenarios prior to experimental and clinical testing. The simulations are part of a feasibility study for the whole approach to ensure higher levels of performance as well as to predict expected issues in real-life configurations.

### 4.2. Fat Thickness

In the presented study, three leg models with varying fat thicknesses were investigated. This allowed to have an overview of the MWT system performance in imaging bones for different scenarios. The investigations carried out in the study suggest that MWT imaging should be applied on body regions with low to medium fat thickness. As seen from the model 1 and model 2 results, bones were detectable for various BVF scenarios. However, in model 3, the reconstructions were not as good. This is due to the thickness of the fat layer as well as the high variations in relative permittivities between consecutive tissue layers (boundaries between skin and far or fat and muscle). For the thick fat scenario, the scattered fields from the bones are highly attenuated before being measured by the receivers; thus, the reconstruction algorithm failed to estimate the bones.

A solution to reduce the effect of fat thickness is the use of prior information about the fat layer that can be included as an input to the inversion algorithm. This will produce satisfactory results as demonstrated in Figure 7. This prior information can be obtained in various way. One technique is to estimate the fat layer thickness using blind inversions, similar to the reconstructions presented in Figure 6 and then to re-run the inversion algorithm with the estimated prior information. The extraction of the fat layer from the blind inversions can be automated using unsupervised clustering techniques. Another method is to estimate the fat thickness using other modalities like MRI or ultrasound; this method might not be cost-effective as there is a need for a second modality. A third technique could be by estimating the fat layer in an ad-hoc manner until satisfactory results are produced.

### 4.3. Number of Antennas

The mathematical problem associated with MWT is an ill-posed non-unique inverse scattering problem. Thus, if insufficient measurements are given to the inversion algorithm, the output reconstructions will not be satisfactory. This have been observed in the reconstructions presented in Figure 13, where the number of antennas are reduced from twenty-four to twelve. Furthermore, having twenty-four transducers in a MWT system is possible by the proper selection of frequency and antenna type. Twenty-four antennas were used successfully in [42,43]. Moreover, based on the investigation presented in Section 3.1.2, the use of eighteen antennas is possible and will produce acceptable results (the tibia bone was reconstructed, which is sufficient to detect BVF variations). This is promising as using less antennas would reduce the complexity of the system as well as reduce the computational time of the inversion algorithm.

### 4.4. Study Limitations

A limitation in this study is the use of two-dimensional (2D) models rather than three-dimensional (3D) models for the leg. Although 3D microwave imaging provides more realistic information for tissue imaging, the proposed 2D MWT system in the study is considered less complex to model, more time efficient for calculations, and less expensive to build as a wearable system for frequent use by doctors and patients. Additionally, inversion algorithms for 3D imaging systems require extensive calculations to perform reconstructions. Further, the use of 2D algorithms to image 3D organs has been performed previously in breast and brain imaging. The 3D effects can be mitigated by using a proper matching medium as well as performing proper calibration on the collected measurements.

Another limitation in this work is that it is based on numerical simulations rather than experimental work. Numerical simulations were considered herein because, as mentioned earlier, it is a feasibility study to illustrate 2D MWT for monitoring variations in bone density. In future works, experimental studies that utilize anatomical phantoms should be performed to take into account realistic factors, such as mutual coupling between antennas, inaccuracies in antenna positions, dynamic range of the system, etc.

A third limitation of this study has to do with the type, size, and number of antennas used. In this paper, there were twenty-four point sources operating at 0.8 GHz. At this selected frequency, the size of the antennas might be large, which might pose challenges in having twenty-four antennas in the imaging chamber. More studies need to be carried out to either increase the frequency (to decrease antenna size), reduce the number of antennas, or both.

Lastly, despite of the good performance in detecting BVF variations from the reconstructed images, the proposed approach, using the expert-eye technique, requires manual tuning of the algorithm. This semi-automatic nature of the segmentation procedure could be enhanced by investigating more automated algorithms, such as unsupervised clustering or supervised learning techniques. Further, while the results obtained were promising in detecting permittivity changes with respect to BVF, more statistical validation is required to assess the technique. This statistical validation could be performed with the inclusion of a large number of samples, which was not performed in this feasibility study.

## 5. Conclusions

In this study, a numerical feasibility study was conducted to evaluate the use of MWT for imaging human bones with varying parameters. The importance of utilizing such systems lies in the ability to perform imaging more frequently, which is due to the non-harmful nature of MWT radiations (low-power non-ionizing electromagnetic waves) when compared to the ionizing X-ray radiation of the current gold standard systems.

In addition, this study proposed the use of an ultrasound gel instead of the more traditional glycerin/water solution as a matching medium for biomedical MWT applications. This allows for the development of wearable, more compact, portable MWT systems in the future because it is more feasible to use a gel instead of a liquid solution as matching medium. The use of ultrasound gel is less complex in cleaning and storage.

Overall, this study paves the way toward implementing a MWT system for bone imaging applications. Although the study was successful in localizing and estimating bones in reconstructed images, further experimental and clinical validations are required, which are considered as a future aspect to the current study. Furthermore, the actual development of a MWT system that could be wearable, especially with the utilization of the ultrasound gel, is promising for many in-home patient management and continuous monitoring procedures.

Lastly, future works can target enhancing the image processing algorithm for automating bone segmentation. A more reliable and automated approach would save post-processing time and assist experts, such as medical doctors, in detecting bones with a low percentage of errors.

## Figures and Tables

**Table 2:** A comparison of the root mean square error (RMSE) values between the actual real-part permittivity values and the extracted values from the reconstructed images for different BVF values.

	Model 1	Model 2
Tibia Bone	4.52	2.54
Fibula Bone	8.56	27.36

## Abbreviations

The following abbreviations are used in this manuscript:

BVF	Bone volume fraction
CSI	Contrast source inversion
DXA	dual-energy X-ray absorptiometry
FEM	Finite-element method
MWT	Microwave tomography
MRI	Magnetic resonance imaging
NOF	National osteoporosis foundation
QCT	Quantitative computed tomography
TX	Transmitters
RX	Receivers
